# Supplementary material for: Genome-wide CRISPR screening identifies Annexin A1 as a facilitator of porcine astrovirus entry
Source: PLoS Pathog. 2026 Feb 2;22(2):e1013943. doi: 10.1371/journal.ppat.1013943 (PMC12880748; doi:10.1371/journal.ppat.1013943)
Supplement: S1 Fig — (A), Multi-step growth curves of PAstV-infected PK15 and IPEC-J2 cells. (B), Cytopathic effects in PK15 cells infected with different MOIs of PAstV. (C), RT-qPCR validation of gene knockdown efficiency using top-ranked enriched sgRNAs or non-targeting control sgRNA. (D), Flow cytometry of PAstV capsid staining in siRNA-transfected PK15 cells, showing the percentage of PAstV⁺ cells for each targeted gene compared with NT. Data represent mean ± SD (n = 3). Statistical analysis was performed by unpaired Two-way ANOVA. (ns, P > 0.05; *P < 0.05; **P < 0.01; ***P < 0.001). (DOCX) [file ppat.1013943.s001.docx]

**

**

**S1 Fig. CRISPR screening identifies ANXA1 as a critical host factor for PAstV infection.**

(A) Multi-step growth curves of PAstV-infected PK15 and IPEC-J2 cells. (B) Cytopathic effects in PK15 cells infected with different MOIs of PAstV. (C) RT-qPCR validation of gene knockdown efficiency using top-ranked enriched sgRNAs or non-targeting control sgRNA. (D), Flow cytometry of PAstV capsid staining in siRNA-transfected PK15 cells, showing the percentage of PAstV⁺ cells for each targeted gene compared with NT. Data represent mean ± SD (n = 3). Statistical analysis was performed by unpaired Two-way ANOVA. (ns, P > 0.05; *P < 0.05; **P < 0.01; ***P < 0.001).
