# Supplementary material for: Genome-wide CRISPR screening identifies Annexin A1 as a facilitator of porcine astrovirus entry
Source: PLoS Pathog. 2026 Feb 2;22(2):e1013943. doi: 10.1371/journal.ppat.1013943 (PMC12880748; doi:10.1371/journal.ppat.1013943)
Supplement: S2 Fig — (A) GFP-positive plasmids used as transfection controls to assess sgRNA packaging efficiency. (B) Sequencing confirmation of ANXA1 knockout (PK15-ANXA1KO) compared to WT cells. (C) Cytopathic effects in PK15-WT and PK15-ANXA1KO cells infected with PAstV (MOI = 1) for 24 h. (D) Virus internalization in PK15 WT and ANXA1 KO. Cells were bound with PAstV at 4°C for 60 min, washed, shifted to 37°C for 30 min, surface virus removed by trypsin, and PAstV genomes quantified by RT-qPCR. Readouts: internalized genomes per well and internalization efficiency, defined as internalized divided by bound. (E) Sequencing result of the ANXA1 rescue plasmid. (F) Preparation and expression of recombinant ANXA1 protein; 1: induced recombinant protein; 2: induced pET-32a vector; 3: non-induced recombinant protein; 4: non-induced pET-32a vector; 5: purified supernatant; 6: purified precipitated protein. (G) Immunofluorescence analysis of PAstV infection (MOI = 0.01) in IPEC-J2-WT and IPEC-J2-ANXA1KO polyclonal knockout cells. Data represent mean ± SD (n = 3). Statistical analysis was performed by unpaired Two-way ANOVA. (ns, P > 0.05; *P < 0.05; **P < 0.01; ***P < 0.001). (DOCX) [file ppat.1013943.s002.docx]

**

**

**S2 Fig. ANXA1 is required for PAstV infection.**

(A) GFP-positive plasmids used as transfection controls to assess sgRNA packaging efficiency. (B) Sequencing confirmation of ANXA1 knockout (PK15-ANXA1KO) compared to WT cells. (C) Cytopathic effects in PK15-WT and PK15-ANXA1KO cells infected with PAstV (MOI=1) for 24 h. (D) Virus internalization in PK15 WT and ANXA1 KO. Cells were bound with PAstV at 4 °C for 60 min, washed, shifted to 37 °C for 30 min, surface virus removed by trypsin, and PAstV genomes quantified by RT-qPCR. Readouts: internalized genomes per well and internalization efficiency, defined as internalized divided by bound. (E) Sequencing result of the ANXA1 rescue plasmid. (F) Preparation and expression of recombinant ANXA1 protein; 1: induced recombinant protein; 2: induced pET-32a vector; 3: non-induced recombinant protein; 4: non-induced pET-32a vector; 5: purified supernatant; 6: purified precipitated protein. (G) Immunofluorescence analysis of PAstV infection (MOI=0.01) in IPEC-J2-WT and IPEC-J2-ANXA1KO polyclonal knockout cells. Data represent mean ± SD (n = 3). Statistical analysis was performed by unpaired Two-way ANOVA. (ns, P > 0.05; *P < 0.05; **P < 0.01; ***P < 0.001).
