# Supplementary material for: Genome-wide CRISPR screening identifies Annexin A1 as a facilitator of porcine astrovirus entry
Source: PLoS Pathog. 2026 Feb 2;22(2):e1013943. doi: 10.1371/journal.ppat.1013943 (PMC12880748; doi:10.1371/journal.ppat.1013943)
Supplement: S3 Fig — (A) Western blot analysis confirming expression of various viral protein constructs in HEK-293T cells. (B) BiFC analysis showing interaction between ANXA1 and ORF2 plasmids. (C) In silico prediction of interaction between ANXA1 and ORF2 proteins. (D) Homology analysis of ANXA1 among porcine, human, and mouse species. (E) PCR results for recombinant ANXA1 plasmid construction using SOE technique. (F) Flow cytometry analysis of PAstV-ILOV infection (MOI = 1) in PK15-ANXA1KO cells transfected with various ANXA1 recombinant plasmids. (DOCX) [file ppat.1013943.s003.docx]

**S3 Fig. ANXA1 directly binds to the PAstV ORF2 acidic domain via its repeat III domain.**

(A) Western blot analysis confirming expression of various viral protein constructs in HEK-293T cells. (B) BiFC analysis showing interaction between ANXA1 and ORF2 plasmids. (C) In silico prediction of interaction between ANXA1 and ORF2 proteins. (D) Homology analysis of ANXA1 among porcine, human, and mouse species. (E) PCR results for recombinant ANXA1 plasmid construction using SOE technique. (F) Flow cytometry analysis of PAstV-ILOV infection (MOI=1) in PK15-ANXA1KO cells transfected with various ANXA1 recombinant plasmids.
