# Supplementary material for: Genome-wide CRISPR screening identifies Annexin A1 as a facilitator of porcine astrovirus entry
Source: PLoS Pathog. 2026 Feb 2;22(2):e1013943. doi: 10.1371/journal.ppat.1013943 (PMC12880748; doi:10.1371/journal.ppat.1013943)
Supplement: S4 Fig — (A) BEV RNA loads at 24 hpi in PK15-WT and PK15-ANXA1KO cells (RT–qPCR, left) and Annexin V/PI staining of early apoptotic cells (Annexin V ⁺ PI ⁻ , right). (B) Gene expression distribution for each RNA-seq sample. (C) Sample-to-sample correlation heatmap. (D) PCA of transcriptomes. E, Summary of DEGs. (F) Volcano plot of DEGs between PAstV-infected PK15-WT and PK15-ANXA1KO cells. (G) GO enrichment for selected DEGs. H, WB of p-IRF3 in PK15-WT and PK15-ANXA1KO cells transfected with 5′ppp-dsRNA. (DOCX) [file ppat.1013943.s004.docx]

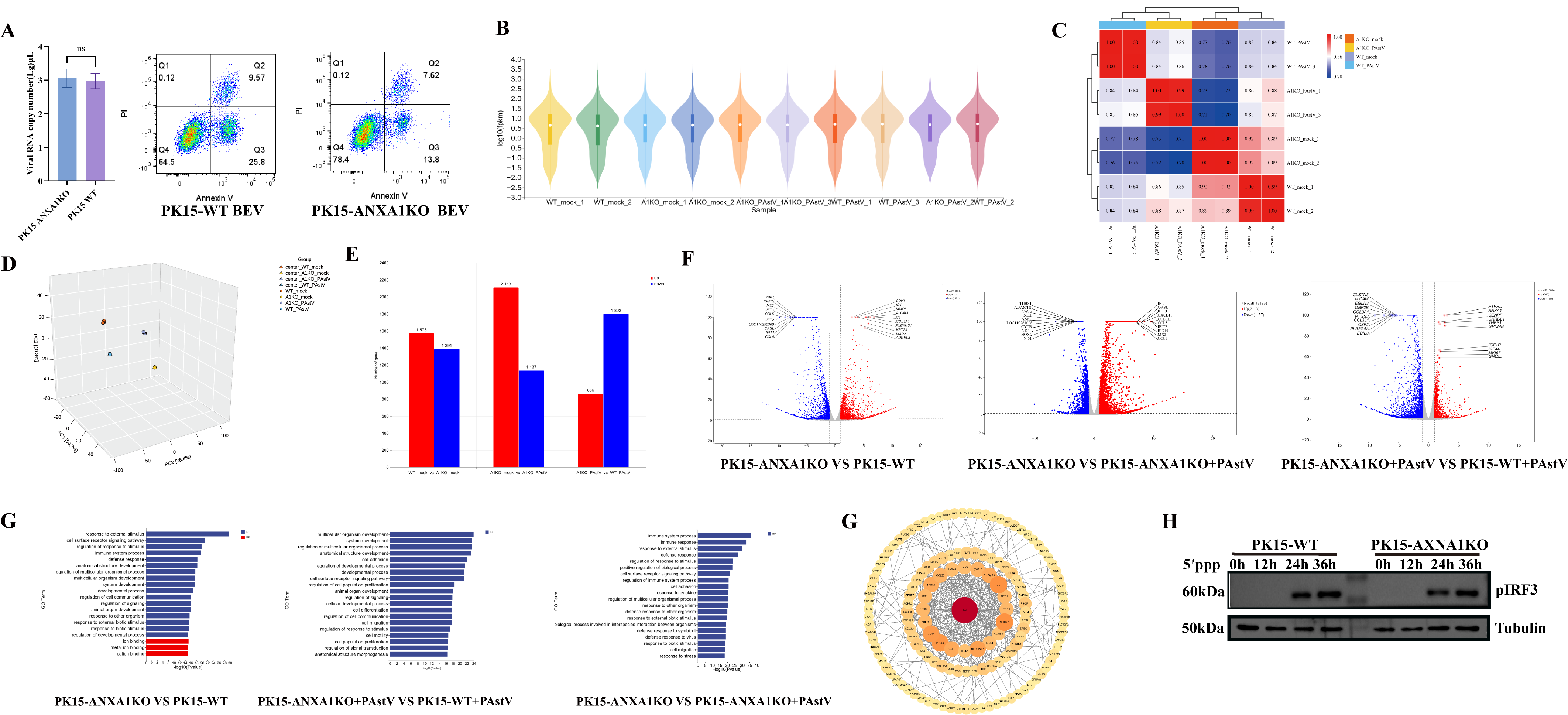


**S4 Fig. ANXA1 modulates virus-induced apoptosis and RIG-I–IRF3 signalling.**

(A) BEV RNA loads at 24 hpi in PK15-WT and PK15-ANXA1KO cells (RT–qPCR, left) and Annexin V/PI staining of early apoptotic cells (Annexin V⁺ PI⁻, right). (B) Gene expression distribution for each RNA-seq sample. (C) Sample-to-sample correlation heatmap. (D) PCA of transcriptomes. E, Summary of DEGs. (F) Volcano plot of DEGs between PAstV-infected PK15-WT and PK15-ANXA1KO cells. (G) GO enrichment for selected DEGs. (H) WB of p-IRF3 in PK15-WT and PK15-ANXA1KO cells transfected with 5′ppp-dsRNA.
