# Supplementary material for: Genome-wide CRISPR screening identifies Annexin A1 as a facilitator of porcine astrovirus entry
Source: PLoS Pathog. 2026 Feb 2;22(2):e1013943. doi: 10.1371/journal.ppat.1013943 (PMC12880748; doi:10.1371/journal.ppat.1013943)
Supplement: Attachment — Submitted filename: Response to Reviewers.docx [file ppat.1013943.s010.docx]

**Editor：**

"Figure 6 (visual summary) still needs work. It is not clear how the "capsid acidic domain" is represented: since neither the main text nor the legend to Figure provide an explanation of exactly what is shown (shapes?, colors?), it is not possible to reconstruct the interaction proposed. The lower part of Figure 6 is also misleading. Since there is no legend, one has to guess what is going on. To my perception the formation of double stranded viral RNA in the cytoplasm is postulated, followed by its transport to the nucleus and possibly even its integration in the genome. Obviously, the second and third step are not known to occur. The authors need to provide a Figure 6 consistent with our current understanding of virus and cell biology, and a detailed explanatory legend, before the manuscript can be accepted." Figure 6 still appears to indicate that the astrovirus RNA integrates in the cellular genome. Moreover, a detailed explanatory legend to Figure 6 is still missing**.**

**Response:** Thank you for the final opportunity to revise. We apologize that our previous submission did not clearly address your comment on Figure 6. We have now substantially revised Figure 6 and added a detailed legend to ensure the schematic is fully interpretable and consistent with established virus and cell biology.

Capsid acidic domain representation: We added an explicit inset that defines the interaction interface, showing that the ANXA1 R3 domain engages the acidic domain within the ORF2 capsid. The ORF2 capsid and its acidic region are clearly labelled in the figure and explained in the legend.

Removal of misleading elements: We redrew the lower panel to avoid any implication that PAstV RNA enters the nucleus or integrates into the host genome. The nucleus is shown only to indicate host IFN-β transcription downstream of IRF3 activation; viral RNA is depicted as remaining in the cytosol.

Legend completeness: We expanded the legend to explain the meaning of the graphical elements (including arrows/dashed lines) so readers do not need to infer shapes or colours

**Reviewer 1**

**Major Issues：**

The authors have addressed my concerns. The titles of the figures remain misleading. ANX1 is not "crucial" f(fig 1) nor "required and sufficient" (Fig 2 and 3) for viral replication.

**Response:** Thank you for your careful check and for pointing this out. We apologize for overlooking these figure titles in the previous revision. We have now revised the titles of Figs. 1–3 to remove potentially misleading wording (e.g., “crucial” and “required and sufficient”) and to better align with the corresponding data and conclusions.

**Reviewer 2**

Figure 6 still depicts an enveloped virus to represent PAstV, a nonenveloped virus, and this should be corrected. Only the icosahedral capsid with ssRNA genome (no outer envelope) should be shown for the virion entering the cell and it should not be fusing with the membrane

**Response:**

Thank you for this important clarification. We have redrawn Fig. 6. We removed any membrane fusion representation to accurately reflect the entry process
